# Realizing Textbook Outcomes Following Liver Resection for Hepatic Neoplasms with Development and Validation of a Predictive Nomogram

**DOI:** 10.1245/s10434-024-15983-6

**Published:** 2024-08-05

**Authors:** Kaival K. Gundavda, Shraddha Patkar, Sadhana Kannan, Gurudutt P. Varty, Kunal Nandy, Tanvi Shah, Kaushik Polusany, Sohan Lal Solanki, Suyash Kulkarni, Nitin Shetty, Kunal Gala, Vikas Ostwal, Anant Ramaswamy, Prabhat Bhargava, Mahesh Goel

**Affiliations:** 1 Department of Gastrointestinal and Hepatobiliary Surgery, Department of Surgical Oncology, Tata Memorial Hospital, Homi Bhabha National Institute (HBNI), Mumbai, Maharashtra India; 2 Department of Biostatistics, The Advanced Centre for Treatment, Research and Education in Cancer (ACTREC), Homi Bhabha National Institute (HBNI), Mumbai, Maharashtra India; 3 Department of Anaesthesiology, Critical Care and Pain, Tata Memorial Hospital, Homi Bhabha National Institute (HBNI), Mumbai, Maharashtra India; 4 Department of Intervention Radiology, Tata Memorial Hospital, Homi Bhabha National Institute (HBNI), Mumbai, Maharashtra India; 5 Department of Medical Oncology, Tata Memorial Hospital, Homi Bhabha National Institute (HBNI), Mumbai, Maharashtra India

**Keywords:** Textbook outcome, Liver resection, Hepatic neoplasms, Nomogram, Overall survival, Low-middle income economy

## Abstract

**Background:**

‘Textbook Outcome’ (TO) represents an effort to define a standardized, composite quality benchmark based on intraoperative and postoperative endpoints. This study aimed to assess the applicability of TO as an outcome measure following liver resection for hepatic neoplasms from a low- to middle-income economy and determine its impact on long-term survival. Based on identified perioperative predictors, we developed and validated a nomogram-based scoring and risk stratification system.

**Methods:**

We retrospectively analyzed patients undergoing curative resections for hepatic neoplasms between 2012 and 2023. Rates of TO were assessed over time and factors associated with achieving a TO were evaluated. Using stepwise regression, a prediction nomogram for achieving TO was established based on perioperative risk factors.

**Results:**

Of the 1018 consecutive patients who underwent liver resections, a TO was achieved in 64.9% (661/1018). The factor most responsible for not achieving TO was significant post-hepatectomy liver failure (22%). Realization of TO was independently associated with improved overall and disease-free survival. On logistic regression, American Society of Anesthesiologists score of 2 (*p* = 0.0002), perihilar cholangiocarcinoma (*p* = 0.011), major hepatectomy (*p* = 0.0006), blood loss >1500 mL (*p* = 0.007), and presence of lymphovascular emboli on pathology (*p* = 0.026) were associated with the non-realization of TO. These independent risk factors were integrated into a nomogram prediction model with the predictive efficiency for TO (area under the curve 75.21%, 95% confidence interval 70.69–79.72%).

**Conclusion:**

TO is a realizable outcome measure and should be adopted. We recommend the use of the nomogram proposed as a convenient tool for patient selection and prognosticating outcomes following hepatectomy.

Liver resection is an established standard treatment for malignant and some benign liver tumors. Despite technological advances and the use of intraoperative adjuncts, liver resection remains technically challenging and is associated with significant morbidity and mortality. Optimizing outcomes following liver resection requires meticulous patient selection, a thorough understanding of the anatomical tenets, and early identification of postoperative complications.^1^

Traditional outcome measures such as the Clavien–Dindo classification (CDC) system, although universally acceptable, are not tailored to the application in the post-hepatectomy setting.^2,3^ The Comprehensive Complication Index (CCI) was proposed to standardize the reporting of surgical complications; however, it was calculated as a sum of all complications weighted for their severity based on the CDC system.^4^ Over a decade ago, the International Study Group of Liver Surgery (ISGLS) proposed a classification of complications unique to hepatobiliary surgery.^5–7^ Despite this, there was an unmet need for a comprehensive and unified system to define the ideal postoperative course after liver resection and to enable standardization and comparison on a global scale. ‘Textbook Outcomes’ (TO﻿) were determined with the objective of hospital quality of care with special attention to patient-centered care, and may represent a more holistic approach to assess and standardize postoperative outcomes.^8^

Although the concept of TO has been used previously in the field of gastrointestinal cancer surgery, it was not until 2023 that an international expert consensus-based definition was proposed, using the formal Delphi approach. Regrettably, only 4.6% of the expert panel members that represented 80 countries classified as lower- to middle-income and low-income economies.^8^

This study aimed to assess the feasibility of including TO as a standard outcome measure following liver resection in a lower- to middle-income country (LMIC). We identified our rates of TO and their temporal trends over a decade, identified factors predicting TO, and determined the impact of TO on overall survival (OS) and disease-free survival (DFS). Based on identified perioperative predictors, we proposed and validated a nomogram-based scoring and risk stratification system to predict the achievement of TO following liver resections.

## Materials and Methods

### Study Population

Data were collected from a prospectively maintained database in the Gastrointestinal and Hepatobiliary Division of the Department of Surgical Oncology. Consecutive patients with suspected hepatic neoplasms who underwent curative resection from January 2011 to June 2023 were included. Analysis for postoperative outcome measures as per the Textbook Outcome in Liver Surgery (TOLS) criteria were analyzed.^8^

The demographic, clinicopathological and operative parameters analyzed were age, sex, body mass index (BMI), American Society of Anesthesiologists (ASA) class, histopathological diagnosis, size and location of the lesion (i.e., unilobar or bilobar), extent of liver resection, surgical approach, intraoperative blood loss, and micro- and macrovascular invasion.

### Inclusion Criteria

All patients with suspected primary hepatic neoplasms, as well as liver secondaries, who underwent resection with curative intent were included. Patients found to have inoperable disease intraoperatively were excluded from the study.

### Preoperative Assessment

The preoperative evaluation included physical examination; contrast-enhanced cross-sectional imaging and relevant tumor markers were assessed. In suspected primary hepatic neoplasms, serum α-fetoprotein (AFP) and protein induced by vitamin K absence-II (*PIVKA*-II) were routinely performed, whereas in the case of secondaries from the gastrointestinal tract, carcinoembryonic antigen (CEA)/carbohydrate antigen 19.9 (CA-19.9) levels were estimated. The preferred initial imaging modality was a triphasic contrast-enhanced computed tomography (CECT) scan of the thorax, abdomen and pelvis. Magnetic resonance imaging (MRI) was used as a troubleshooting modality, especially in cirrhotic livers. A percutaneous biopsy was performed selectively, and in patients who had undergone a biopsy elsewhere, a pathology review was obtained.

### Treatment

All patients were discussed in a dedicated hepatobiliary multidisciplinary tumor board, comprising a hepatobiliary surgical oncologist, intervention radiologist, medical oncologist, radiation oncologist, and pathologist. Patients deemed suitable for surgery underwent a thorough preoperative anesthesia assessment, nutritional rehabilitation, and chest physiotherapy evaluation.

In patients planned for a major hepatectomy, defined as the resection of three or more Couinaud segments,^9^ the future functional liver remnant (FLR) was confirmed by volumetric assessment on radiology and an indocyanine green retention (ICG -R15) test.

Liver resections were performed using a combination of transection devices, such as a cavitron ultrasonic surgical aspirator (CUSA), waterjet system, procoagulators, and coagulating devices, such as bipolar forceps and vessel sealant, without routine use of vascular occlusion. Enhanced recovery after surgery (ERAS) protocols were practised during the perioperative period.^10^ Avoidance of volume overload was ensured to reduce blood loss, by central venous pressure monitoring, goal-directed fluid therapy, use of epidural analgesia and acute normovolemic intraoperative hemodilution. Early oral intake and mobilization were encouraged in the postoperative period, supplemented with the use of multimodal analgesia.

### Pathology Review

Pathology review included confirmation of the primary diagnosis, tumor location, tumor size, histological differentiation, lymphovascular invasion (LVI), perineural invasion (PNI), and resection margins. By the TOLS definition, a 1 mm parenchymal margin was sufficient to categorize as R0 resection.^8^

### Post-Treatment Surveillance

Follow-up was performed on an outpatient basis with liver function tests, disease-specific tumor markers, and transabdominal ultrasonography every three months for 2 years. After 2 years, patients were followed up twice yearly, and then annually after the 5th year. A CECT of the thorax and abdomen was performed alternately with an ultrasound at intervals of 3–6 months for the first 2 years, then 6-monthly for 3 years, and then annually up to 5 years or whenever there was a suspicion of relapse.

### Definitions and Data Collection

Textbook outcomes (TO) were defined as per the consensus, after consideration of the following parameters:^8^ (1) absence of intraoperative grade ≥2 incidents scored according to the Oslo classification;^11^ (2) absence of postoperative complications graded according to the CDC system, with CD grade IIIa or higher being considered a major complication;^3^ (3) absence of postoperative bile leaks and post-hepatectomy liver failure, defined by the ISGLS;^5,6^ (4) negative resection margins, defined as R0 (1 mm or more tumor-free margin), R1 (<1 mm tumor-free margin), or R2 (macroscopic tumor involvement at the margin); (5) readmission, defined as unplanned rehospitalization within 90 days after discharge; and (6) mortality, defined as death due to any cause in-hospital or within 90 days of the surgical procedure.

### Statistical Analysis

Continuous variables were described as median (interquartile range [IQR]) or mean (standard deviation [SD]), while categorical variables were described as number (%). Differences in baseline characteristics were assessed using the Kruskal–Wallis test for continuous variables and Chi-square or Fisher’s exact tests for categorical variables.

A univariate logistic regression model was used to test the ability of potential baseline risk factors to predict the risk of TOLS. Significant variables were further entered into the multivariate logistic regression analysis to identify independent prognostic factors. The whole cohort was randomly assigned to define development and validation cohorts. The reliability of the predictive model was assessed with respect to discrimination and calibration. Predictive model discrimination was analyzed by the area under the receiver operating characteristic (ROC) curve. The model’s calibration was tested using a calibration plot and performance was evaluated using the concordance index.

The training and test cohorts were divided into high- and low-risk scores based on the median of the total nomogram score. Factors differentiating the risk groups were compared using the Chi-square test for the training and test cohorts. Kaplan–Meier curves were used to compare the survival probabilities between the high- and low-risk groups.

All statistical analyses were performed using Statistical Product and Service Solutions (SPSS) version 26 (IBM Corporation, Armonk, NY, USA), and a *p*-value <0.05 was considered statistically significant.

## Results

### Clinicopathologic Demographics

A total of 1018 consecutive patients of suspected hepatic neoplasms undergoing curative resections between January 2011 and June 2023 were included in the cohort. The median patient age was 54 years (IQR 43–63), with almost two-thirds of patients being male (*n* = 668, 65.6%). The most common indication for surgery was primary hepatic neoplasms (*n* = 519, 51%), followed by colorectal liver metastasis (CRLM) in one-quarter of the patients (*n* = 252, 24.8%). The majority of patients were ASA class 1 (*n* = 523, 51.4%) and underwent a major liver resection (*n* = 531, 52.2%), predominantly via an open surgical approach (*n* = 972, 95.5%).

The demographic, clinicopathologic, and treatment characteristics of the analytic cohort and training and test cohorts are summarized in Table 1.

**Table 1:** Comparison of demographic, tumor, and treatment characteristics and immediate postoperative outcomes

Variable	All patients [*n *= 1018]	Training cohort [*n *= 455]	Test cohort [*n *= 194]	*p*-Value
*Demographics*				
Age at diagnosis, years [median (IQR)]	54 (43, 63)	56 (45, 64)	53.5 (44, 62)	0.393
Sex				
Male	668 (65.6)	305 (67)	130 (67)	1
Female	350 (34.4)	150 (33)	64 (33)	
ASA				0.117
1	523 (51.4)	200 (44)	92 (47.4)	
2	442 (43.4)	227 (49.9)	83 (42.8)	
3 and 4	53 (5.2)	28 (6.2)	19 (9.8)	
*Tumor characteristics*				
Diagnosis				0.2896
Primary hepatic neoplasm	519 (51)	237 (52.1)	97 (50)	
CRLM	252 (24.8)	116 (25.5)	45 (23.2)	
NET mets	61 (6)	35 (7.7)	12 (6.2)	
pHCC	55 (5.4)	27 (5.9)	12 (6.2)	
Others	131 (12.9)	40 (8.8)	28 (14.4)	
* T*_max_, cm [median (IQR)]	5.5 (3.2–9)			
Lymphovascular invasion				0.52
No	682 (66.9)	338 (74.4)	139 (71.6)	
Yes	212 (20.85)	116 (25.6)	55 (28.4)	
NK	124 (12.1)	1	0	
Margins				
Free (R0)	970 (95.3)	431 (94.7)	187 (96.4)	0.4775
Positive	48 (4.7)	24 (5.3)	7 (3.6)	
*Treatment characteristics*				
Extent of resection				0.2052
Major	531 (52.2)	234 (51.4)	111 (57.2)	
Minor	487 (47.8)	221 (48.6)	83 (42.8)	
Surgical approach				0.6291
Open	972 (95.5)	429 (94.3)	182 (93.8)	
Laparoscopic	23 (2.3)	11 (2.4)	7 (3.6)	
Robotic	23 (2.3)	15 (3.3)	5 (2.6)	
Surgery duration, min [median (IQR)]	240 (180–320)	240 (170–320)	240 (180–300)	0.1453
*Immediate postoperative outcomes*				
Significant PHLF				0.4553
No	794 (78)	345 (75.8)	141 (72.7)	
Yes	224 (22)	110 (24.2)	53 (27.3)	
Significant PHBL				0.3505
No	944 (92.7)	422 (92.7)	175 (90.2)	
Yes	74 (7.3)	33 (7.3)	19 (9.8)	
Significant complication				1
No	815 (80.1)	360 (79.1)	154 (79.4)	
Yes	203 (19.9)	95 (20.9)	40 (20.6)	
Mortality				0.3011
No	970 (95.3)	437 (96)	182 (93.8)	
Yes	48 (4.7)	18 (4)	12 (6.3)	
Readmission				0.1559
No	974 (95.7)	436 (95.8)	180 (92.8)	
Yes	44 (4.3)	19 (4.2)	14 (7.2)	

Data are expressed as *n* (%) unless otherwise specified

*ASA* American Society of Anesthesiologists, *NK* not known, *IQR* interquartile range, *CRLM* colorectal liver metastasis, *NET* neuroendocrine tumor, *pHCC* perihilar cholangiocarcinoma, *T*_max_ maximum tumor dimension, *PHLF* post-hepatectomy liver failure, *PHBL* post-hepatectomy bile leak, *mets* metastasis

### Textbook Outcome Rates and Trends Over Time

TO were achieved in 64.9% (661/1018) of cases. Post-hepatectomy liver failure (*n* = 224, 22%), followed by significant complications (*n* = 203, 19.9%), had the greatest negative impact on the ability to obtain a TO. Absence of readmission (*n* = 974, 95.7%) and no mortality (*n* = 970, 95.3%) were the outcomes most commonly realized, followed by a margin-negative resection (*n* = 970, 95.3%).

When stratified by extent of liver resection, TO was seen in 51% of major resections (271/531) and 80.1% of minor resections (390/487).

The incidence of TO ranged from 54% to 70% across the study period, with no significant increase in trends over time (*p*_trend_ = 0.09).

### Impact of Textbook Outcomes (TO) on Long-Term Survivals

**Fig. 1:**
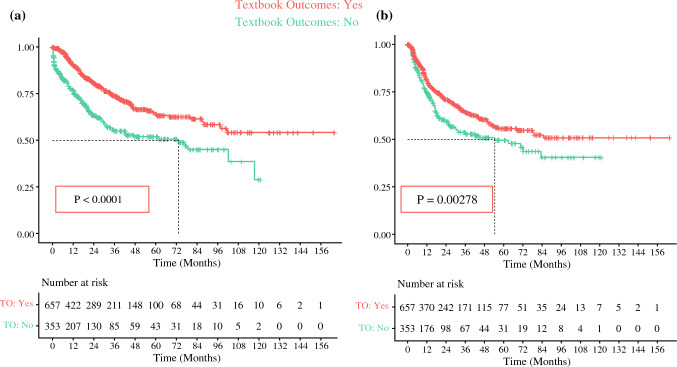
Kaplan–Meier curves depicting **a** overall survival and **b** disease-free survival between patients achieving a TO versus no TO. *TO* Textbook Outcome

TO was significantly associated with improved OS, even after adjustment on multivariate analysis (*p* < 0.001). Estimated survival for patients with TO at 3 years was 74% (69.8–78.5%) versus 55.2% (49.1–62%) for patients not achieving TO (*p* = 0.001); at 5 years, estimated survival was 64.8% (59.7–70.4%) and 52% (45.7–59.2%), respectively (Fig. 1a)

The estimated DFS for TO at 3 years was 64.5% (60–69.4%) versus 53.9% (47.4–61.2%) for patients not achieving TO; at 5 years, the estimated DFS was 55.9% (50.5–61.8%) and 49.7% (42.7–57.8%), respectively (*p* = 0.0028) (Fig. 1b)

### Development and Validation of a Prediction Model for Predicting TO

Several factors were identified on univariate analysis that were associated with TO. On multivariable analysis, patients with perihilar cholangiocarcinoma (pHCC; hazard ratio [HR] 2.9, 95% confidence interval [CI] 1.274–6.642; *p* = 0.011) and presence of lymphovascular emboli (LVE) on pathology (HR 1.587, 95% CI 1.058–2.381; *p* = 0.026) were associated with lesser TO. Major resections (HR 2.454, 95% CI 1.638–3.676; *p* = 0.0006) and blood loss >1500 mL (HR 2.101, 95% CI 1.227–3.597; *p* = 0.007) were also significantly associated with lower TO rates (Table 2)

**Table 2:** Univariate and multivariable analysis of factors predicting realization of Textbook Outcomes following liver resection

Variable		*N*	Univariable		Multivariable	
			OR (95% CI)	*p*-Value	OR (95% CI)	*p*-Value
Age			1.017 (1.007–1.026)	**0.0001**	1.008 (0.994–1.022)	0.293
Sex	Male	668	**1**			
	Female	350	0.693 (0.525–0.915)	**0.010**	0.978 (0.669–1.43)	0.910
ASA	1	523	1	**0.0002**		
	2	442	2.022 (1.545–2.646)	**0.0004**	1.998 (1.362–2.931)	**0.0002**
	3–4	53	2.037 (1.145–3.625)	**0.015**	1.989 (0.962–4.112)	0.063
Histological diagnosis	Primary hepatic neoplasms	519	1			
	CRLM	252	0.752 (0.544–1.04)	0.085	1.64 (1.027–2.621)	0.038
	NET mets	61	0.803 (0.454–1.421)	0.452	0.896 (0.433–1.853)	0.767
	pHCC	55	4.734 (2.547–8.8)	**0.0008**	2.909 (1.274–6.642)	**0.011**
	Others	131	0.673 (0.441–1.027)	0.067	1.082 (0.597–1.961)	0.795
No. of lesions	1	788	0.597 (0.294–1.215)	0.155		
	2	119	0.472 (0.213–1.049)	0.066		
	3	46	0.872 (0.352–2.158)	0.767		
	4	12	0.567 (0.142–2.267)	0.422		
	≥5	18	1.133 (0.357–3.6)	0.832		
Size of largest lesion, cm	<3	239	1			
	3–5	226	1.198 (0.815–1.763)	0.359		
	5–10	321	1.221 (0.855–1.743)	0.272		
	10–20	186	1.12 (0.744–1.685)	0.587		
	≥20	15	4.373 (1.445–13.24)	**0.009**		
Distribution of lesions	Unilobar	908	1			
	Bilobar	75	0.825 (0.495–1.372)	0.458		
LVE	No	682	1			
	Yes	212	1.9 (1.388–2.602)	**0.0005**	1.587 (1.058–2.381)	**0.026**
Surgical approach	Open	972	1			
	Laparoscopic	23	0.08 (0.011–0.599)	**0.014**	0 (0–0)	0.999
	Robotic	23	0.491 (0.181–1.335)	0.164	0.661 (0.164–2.666)	0.561
Extent of resection	Minor hepatectomy	487	1			
	Major hepatectomy	531	3.857 (2.915–5.104)	**0.0002**	2.454 (1.638–3.676)	**0.0006**
Blood loss, mL	<800	219	1			
	800–1500	215	2.422 (1.551–3.784)	**0.0004**	1.389 (0.824–2.34)	0.217
	1500–2550	216	4.448 (2.873–6.885)	**0.0003**	2.101 (1.227–3.597)	**0.007**
	>2550	97	7.166 (4.198–12.233)	**0.0006**	2.677 (1.375–5.209)	**0.004**
Duration of surgery			1.006 (1.004–1.007)	**0.0007**		
Additional organ surgery	No	215	1			
	Yes	803	1.01 (0.737–1.385)	0.949		

*ASA* American Society of Anesthesiologists, *CRLM* colorectal liver metastasis, *NET* neuroendocrine tumor, *pHCC* perihilar cholangiocarcinoma, *LVE* lymphovascular emboli, *OR* odds ratio, *CI* confidence interval, *mets* metastasis

Bold indicate significant *p* values

**Fig. 2:**
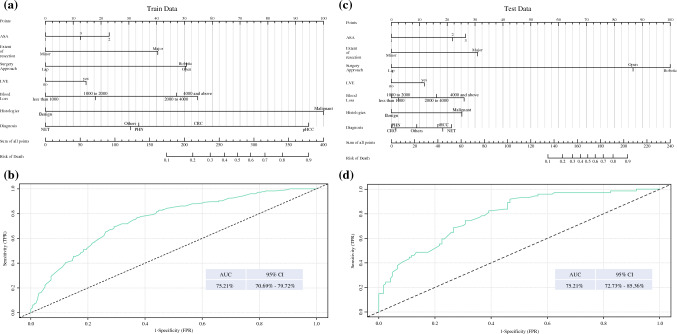
A nomogram model for preoperative estimation of the probability of achieving a TO in the **a** training dataset and **c** test dataset, and performance of the nomogram in predicting TO in the **b** training cohort and **d** test cohort. *ASA* American Society of Anesthesiologists, *AUC* area under the curve, *CI* confidence interval, *CRC* colorectal cancer, *Lap* laparoscopic, *LVE* lymphovascular emboli, *NET* neuroendocrine tumor, *PHN* primary hepatic neoplasms, *pHCC* perihilar cholangiocarcinoma, *TO* Textbook Outcome, *TPR* true positive rate

These independent predictors were integrated into a nomogram prediction model to show the probability of achieving TO, and the points of each factor were calculated by their weight of odds ratios (ORs). Based on the nomogram, the ROC curve analysis was applied to evaluate the accuracy of the prediction. The area under the curve (AUC) of the nomogram model in predicting TO was 75.21% (95% CI 73.14–80.41%). The dataset was divided into a training set and a test set to internally validate the predictive performance of the nomogram model. The AUC of the nomogram model in predicting TO was 75.21% (95% CI 70.69–79.72%) and 75.21% (95% CI 72.73–85.36%) for the training and test sets, respectively (Fig. 2).

**Fig. 3:**
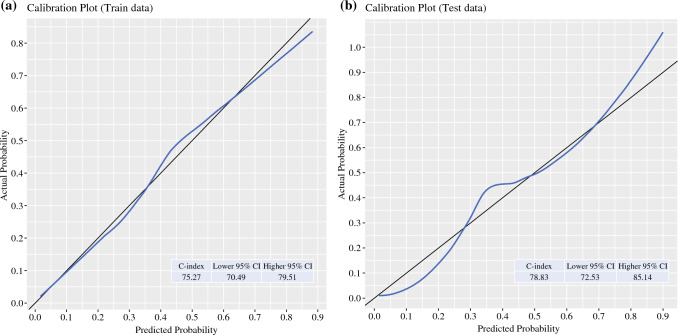
Calibration curve of the nomogram for the estimation of the risk of realizing a Textbook Outcome in the **a** training data and **b** test data. *CI* confidence interval

Calibration curves of the nomogram by bootstrapping showed good agreement between the predicted probability of TO and the actual observed incidence of TO for the training and test cohorts (Fig. 3)

### Performance of the Nomogram

An optimal cut-off value of the nomogram for discriminating TO was identified as 247, based on the median of the total nomogram score. According to this optimal cut-off value of the nomogram score, patients in the test cohort were divided into the following groups: the high-probability TO group (*n* = 328) and the low-probability TO group (*n* = 321).

**Fig. 4:**
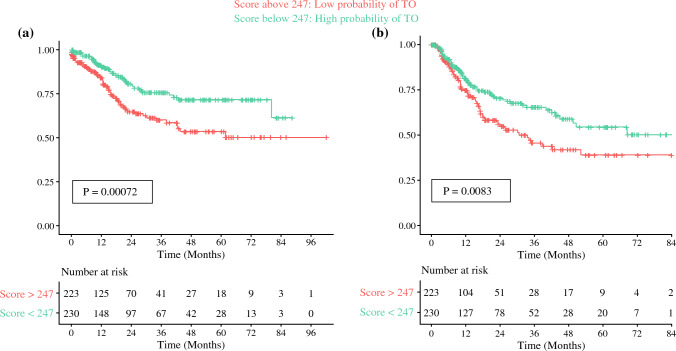
Kaplan–Meier curves comparing **a** overall survival and **b** disease-free survival between patients in the ‘low-probability’ and ‘high-probability’ TO groups. *TO* Textbook Outcome

Patients in the high-probability TO group were confirmed to have a better OS and DFS compared with the low-probability TO group (Fig. 4). The characteristics of patients based on nomogram scores and their postoperative outcomes are summarized in Table 3.

**Table 3:** Comparison of characteristics of patients with nomogram scores: high probability (score <247) and low probability (score >247) of TO in the training set

Variable	Nomogram score		
	High probability of TO (below median) [*n* = 328]	Low probability of TO (above median) [*n* = 321]	*p*-Value
*Treatment characteristics*			
Extent of resection			
Major	62 (18.9)	283 (88.2)	**< 0.001**
Minor	266 (81.1)	38 (11.8)	
Blood loss [median (IQR)]	900 (500, 1500)	2400 (1500, 3525)	**< 0.001**
Surgery duration [median (IQR)]	180 (120, 260)	300 (210, 360)	**< 0.001**
R0 margins			
Yes	315 (96)	303 (94.4)	0.42
No	13 (4)	18 (5.6)	
*Immediate postoperative outcomes*			
Significant PHLF			
Yes	23 (7)	140 (43.6)	**< 0.001**
No	305 (93)	181 (56.4)	
Significant PHBL			
Yes	11 (3.4)	41 (12.8)	**< 0.001**
No	317 (96.6)	280 (87.2)	
Significant complications			
Yes	33 (10.1)	102 (31.8)	**< 0.001**
No	295 (89.9)	219 (68.2)	
Mortality			
Yes	4 (1.2)	26 (8.1)	**< 0.001**
No	324 (98.8)	295 (91.9)	
Long-term outcomes			
Median OS (IQR)	18.7 (7.9, 36.7)	13.2 (5.0, 27.3)	**0.0014**
Median DFS (IQR)	13.4 (6.1, 31.2)	10.1 (3.9, 22.7)	**0.0008**

Data are expressed as *n* (%) unless otherwise specified

*IQR* interquartile range, *PHLF* post-hepatectomy liver failure, *PHBL* post-hepatectomy bile leak, *OS* overall survival, *DFS* disease-free survival, *TO* Textbook Outcome

Bold indicate significant *p* values

## Discussion

Liver resection remains a technically challenging surgical procedure, with morbidity ranging from 20 to 40%, with a mortality rate of <5%.^12,13^ Previous efforts to standardize liver resections in India have reported comparable morbidity and mortality rates.^14^ In recent years, advancements in techniques such as parenchyma preservation,^15^ intraoperative image guidance,^16^ minimal access surgery,^17^ and the implementation of ERAS protocol have improved the safety of liver resections.^10,18^ Despite this, there remained an unmet need for a standardized outcome measure encompassing complex surgical processes’ multidimensional aspects into a single indicator.^8,19^ Although objective and reproducible, the CDC and CCI scoring systems are generally more suitable for heterogeneous populations.^3,4^ The ISGLS subsequently defined and classified intervention-specific complications in patients undergoing hepatobiliary resections.^5–7^

The ‘Textbook Outcome’ was developed as a measure to unify these systems into a single comprehensive indicator of multidimensional and holistic postoperative care and has been used to compare the overall quality of cancer care across hospitals.^20^ Over a span of 12 years, in our experience of 1018 hepatic resections, Textbook Outcomes were achieved in 64.9% (661/1018). These TO rates are comparable with the available literature, with TO rates ranging from as low as 11.2% to upwards of 80% in specialized centers.^19,21–29^

Based on the surgical approach, TO was achieved in 74.8% of patients undergoing laparoscopic liver resection, compared with 61.9% for open surgery.^24^ When categorized by histology, the TO rates for benign, CRLM, and other malignancies were 82%, 73%, and 74%, respectively.^25,29^ However, TO rates for hepatic resection for cholangiocarcinoma are significantly less promising, with a multi-institutional study reporting a TO in only 26% of patients with intrahepatic cholangiocarcinoma.^21,24^ We found that hepatic resections for pHCC were associated with worse outcomes, with TO rates as low as 27.27%. Another study of over 200 resections for pHCC corroborates our poor outcomes.^30^

The analysis of temporal trends of TO across the study period showed that TO ranged from 54% to 70%, and there was no significant increase in trends over time (*p*_trend_ = 0.09). These consistent rates reflect our efforts to standardize liver resections over the past decade.^14^ In another study mirroring our results, no increasing trend in TO rates was found in either major or minor liver resections over a 12-year period.^23^

TO was significantly associated with improved OS and DFS. Multiple studies have similarly reported a significant improvement in long-term OS and DFS associated with TO fulfilment.^23,28–32^ On the other hand, a pairwise comparison demonstrated that the long-term survival following hepatocellular carcinoma (HCC) resection was influenced more by hospital case volume rather than by TO.^33^ In our study, post-hepatectomy liver failure (*n* = 224, 22%), followed by significant complications (*n* = 203, 19.9%), had the greatest negative impact on the ability to obtain a TO, whereas in other studies, the major deterrents to achieving a TO were prolonged length of hospital stay^19^ and major morbidity (Clavien–Dindo ≥3).^29^

Numerous studies have analyzed factors predictive of TO. For open resections, only histological diagnosis of cholangiocarcinoma and a tumor size ≥30 mm were associated with a worse TO rate.^24^ Among patients with pHCC, preoperative biliary drainage, high prognostic nutritional index, and minimally invasive approach were identified as independent predictors of TO, whereas a high ASA score decreased the odds of TO.^30^ Advanced age, concomitant surgery, prolonged operative time, and increased blood loss are associated with lower TO rates.^26,29^ A high tumor burden,^27^ high aspartate transaminase-platelet ratio index (APRI),^27^ Charlson Comorbidity Index >3, major liver resection, intermediate HCC, and a Model for End-Stage Liver Disease (MELD) score >10 are independently associated with not achieving a TO.^27,29^ In another study, the absence of diabetes mellitus (DM) and thrombocytopenia were found to adversely predict TO.^28^ Higher ASA grade was also found to be detrimental to achieving a TO.^29^ Patients undergoing liver surgery at dedicated cancer centers had 31% and 36% higher odds of achieving TO compared with National Cancer Institute-affiliated cancer centers and other US hospitals, respectively.^34^ Extremes of BMI were independently associated with higher incidences of non-TO compared with normal BMI individuals.^35^ Obesity was a factor in failing to achieve TO even after laparoscopic resections.^36^

We noticed that while there are various studies that have developed predictive nomograms for post-hepatectomy liver failure,^37–43^ there is a dearth of predictive models to assess the likelihood of achieving TO. Therefore, we developed a nomogram and used an internal cohort for its validation with good accuracy in predicting TO. A previous study of 687 patients also showed similar predictive probability in both the training set (AUC 0.755) and validation set (AUC 0.763).^21^

The proposed nomogram model enables us to identify patients at high risk of non-realization of TO. This information can guide surgeons to make personalized decisions, thereby enabling an ‘ideal’ postoperative course and ultimately improving long-term survival.^44^ Risk scoring based on perioperative factors may enhance case selection, facilitate close monitoring, and potentially enable timely intervention.

Our study represents the largest experience of assessment of TO in hepatic resections from a single center in South Asia. It allows us to compare immediate postoperative and long-term outcomes among patients who realize a TO against those who do not. Being a high-volume center for liver resections, all treatments were standardized and administered following a multidisciplinary tumor board discussion. Despite operating in resource-constrained settings, from an LMIC, we report outcomes comparable with global standards. This encourages the adoption of TO as a standard outcome measure even in resource-limited settings. Our nomogram utilizes accessible perioperative variables and proposes an effortless scoring system, thereby enabling its widespread implementation in clinical practice. However, the study is limited by its retrospective design and shortcomings of data maintenance in terms of missing data and a 6% loss of follow-up.

## Conclusion

Textbook Outcome after liver resection is a realizable outcome measure and should be adopted even in resource-constrained settings. By incorporating essential perioperative parameters, we developed and validated a simplified nomogram for the prediction of TO that showed good performance in predicting TO after liver resection. We recommend use of the nomogram proposed as a convenient tool for patient selection and prognosticating outcomes following hepatectomy.

## Data Availability

The datasets generated and/or analyzed during the current study are available from the corresponding author upon reasonable request.
